# 2,2′-Dithio­diterephthalic acid

**DOI:** 10.1107/S1600536809013622

**Published:** 2009-04-22

**Authors:** Ling Zhang

**Affiliations:** a Department of Chemistry, Lishui University, 323000 Lishui, ZheJiang, People’s Republic of China

## Abstract

In the title mol­ecule, C_16_H_10_O_8_S_2_, the two aromatic rings form a dihedral angle of 87.97 (12)°. In the crystal structure, inter­molecular O—H⋯O hydrogen bonds [O⋯O = 2.623 (3)–2.639 (3) Å] link the mol­ecules into layers parallel to the *ab* plane.

## Related literature

For complexes of disulfide derivatives, see Li *et al.* (2008 ▶).
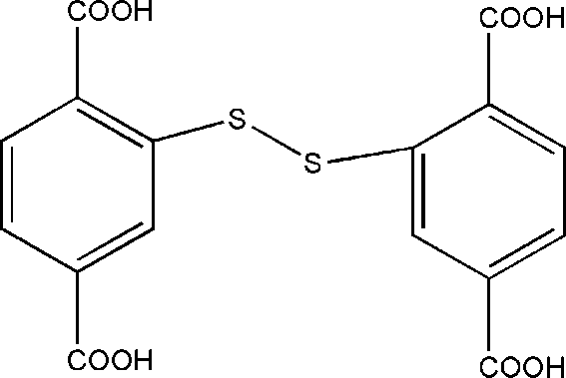


## Experimental

### 

#### Crystal data

C_16_H_10_O_8_S_2_
*M* *_r_* = 394.36
Monoclinic, 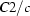

*a* = 16.396 (3) Å
*b* = 9.8462 (15) Å
*c* = 20.363 (3) Å
β = 98.840 (2)°
*V* = 3248.2 (9) Å^3^
*Z* = 8
Mo *K*α radiation
μ = 0.37 mm^−1^
*T* = 298 K
0.48 × 0.21 × 0.03 mm

#### Data collection

Bruker APEXII area-detector diffractometer
Absorption correction: multi-scan (*SADABS*; Sheldrick, 2004 ▶) *T*_min_ = 0.831, *T*_max_ = 0.988
11095 measured reflections
3027 independent reflections
1992 reflections with *I* > 2σ(*I*)
*R*_int_ = 0.039

#### Refinement

*R*[*F* ^2^ > 2σ(*F* ^2^)] = 0.047
*wR*(*F* ^2^) = 0.140
*S* = 1.03
3027 reflections
239 parameters
H-atom parameters constrained
Δρ_max_ = 0.53 e Å^−3^
Δρ_min_ = −0.32 e Å^−3^

### 

Data collection: *APEX2* (Bruker, 2004 ▶); cell refinement: *SAINT* (Bruker, 2004 ▶); data reduction: *SAINT*; program(s) used to solve structure: *SHELXS97* (Sheldrick, 2008 ▶); program(s) used to refine structure: *SHELXL97* (Sheldrick, 2008 ▶); molecular graphics: *ORTEPIII* (Burnett & Johnson, 1996 ▶) and *ORTEP-3 for Windows* (Farrugia, 1997 ▶); software used to prepare material for publication: *SHELXL97*.

## Supplementary Material

Crystal structure: contains datablocks I, global. DOI: 10.1107/S1600536809013622/cv2546sup1.cif

Structure factors: contains datablocks I. DOI: 10.1107/S1600536809013622/cv2546Isup2.hkl

Additional supplementary materials:  crystallographic information; 3D view; checkCIF report

## Figures and Tables

**Table 1:** Hydrogen-bond geometry (Å, °)

*D*—H⋯*A*	*D*—H	H⋯*A*	*D*⋯*A*	*D*—H⋯*A*
O8—H8*D*⋯O5^i^	0.82	1.81	2.632 (3)	174
O6—H6*D*⋯O7^ii^	0.82	1.83	2.633 (3)	166
O3—H3*D*⋯O1^iii^	0.82	1.81	2.623 (3)	174
O2—H2*D*⋯O4^iv^	0.82	1.82	2.639 (3)	174

Symmetry codes: (i) 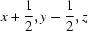; (ii) 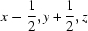; (iii) 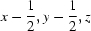; (iv) 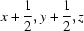.

**Fig. 1.**
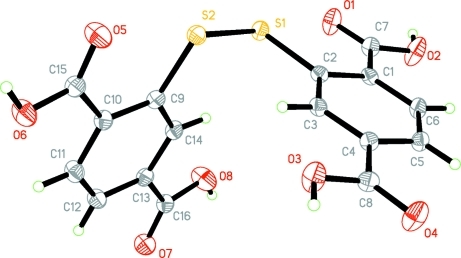
Molecular structure of (I), showing the atom-labelling scheme. Displacement ellipsoids are drawn at the 30% probability level. H atoms are represented as small spheres of arbitrary radii.

## supplementary crystallographic information

### Comment

The disulfide derivatives of the nicotinate - dithiodinicotinates - adopt usually a twisted structure with the C—S—S—C torsion of ca 90° in the solid state, that provides a possibility to show the axial chirality with M- and P-forms of the enantiomers (Li *et al.*,  2008). Herewith we present the crystal structure of the title compound (Fig. 1), where torsion angle C—S—S—C is 91.80 (15)°.

In the crystal, intermolecular O—H···O hydrogen bonds (Table 1) link the molecules into layers parallel to *ab* plane.

### Experimental

2,2'-Disulfanediylditerephthalic acid (0.40 mg, 0.1 mmol), Mn(CH3COO)2 (0.28 mg, 0.11 mmol), NaOH (25 mg, 0.06 mmol) were added in methanol. The mixture was heated and stirred for six hours under reflux. The resultant was then filtered off to give a pure solution which was treated by diethyl ether in a closed vessel. One week later, single crystals were obtained.

### Refinement

All H atoms attached to C atoms or O atoms were fixed geometrically and treated as riding with C—H = 0.93 Å (aromatic) or O—H = 0.82 Å (hydroxyl group) with U_iso_(H) = 1.2U_eq_.

### Crystal data

**Table:**

C_16_H_10_O_8_S_2_	*F*(000) = 1616
*M**_r_* = 394.36	*D*_x_ = 1.613 Mg m^−^^3^
Monoclinic, *C*2/*c*	Mo *K*α radiation, λ = 0.71073 Å
Hall symbol: -C 2yc	Cell parameters from 1947 reflections
*a* = 16.396 (3) Å	θ = 2.4–25.6°
*b* = 9.8462 (15) Å	µ = 0.37 mm^−^^1^
*c* = 20.363 (3) Å	*T* = 298 K
β = 98.840 (2)°	Block, colourless
*V* = 3248.2 (9) Å^3^	0.48 × 0.21 × 0.03 mm
*Z* = 8	

### Data collection

**Table:**

Bruker APEXII area-detector diffractometer	3027 independent reflections
Radiation source: fine-focus sealed tube	1992 reflections with *I* > 2σ(*I*)
graphite	*R*_int_ = 0.039
φ and ω scans	θ_max_ = 25.5°, θ_min_ = 2.4°
Absorption correction: multi-scan (*SADABS*; Sheldrick, 2004)	*h* = −19→19
*T*_min_ = 0.831, *T*_max_ = 0.988	*k* = −11→11
11095 measured reflections	*l* = −24→24

### Refinement

**Table:**

Refinement on *F*^2^	Primary atom site location: structure-invariant direct methods
Least-squares matrix: full	Secondary atom site location: difference Fourier map
*R*[*F*^2^ > 2σ(*F*^2^)] = 0.047	Hydrogen site location: inferred from neighbouring sites
*wR*(*F*^2^) = 0.140	H-atom parameters constrained
*S* = 1.03	*w* = 1/[σ^2^(*F*_o_^2^) + (0.0679*P*)^2^ + 4.4093*P*] where *P* = (*F*_o_^2^ + 2*F*_c_^2^)/3
3027 reflections	(Δ/σ)_max_ < 0.001
239 parameters	Δρ_max_ = 0.53 e Å^−^^3^
0 restraints	Δρ_min_ = −0.32 e Å^−^^3^

### Special details

**Table:**

Geometry. All esds (except the esd in the dihedral angle between two l.s. planes) are estimated using the full covariance matrix. The cell esds are taken into account individually in the estimation of esds in distances, angles and torsion angles; correlations between esds in cell parameters are only used when they are defined by crystal symmetry. An approximate (isotropic) treatment of cell esds is used for estimating esds involving l.s. planes.
Refinement. Refinement of F^2^ against ALL reflections. The weighted R-factor wR and goodness of fit S are based on F^2^, conventional R-factors R are based on F, with F set to zero for negative F^2^. The threshold expression of F^2^ > 2sigma(F^2^) is used only for calculating R-factors(gt) etc. and is not relevant to the choice of reflections for refinement. R-factors based on F^2^ are statistically about twice as large as those based on F, and R- factors based on ALL data will be even larger.

### Fractional atomic coordinates and isotropic or equivalent isotropic displacement parameters (Å^2^)

**Table:**

	*x*	*y*	*z*	*U*_iso_*/*U*_eq_	
S1	0.26721 (5)	1.00797 (9)	0.25924 (4)	0.0312 (2)	
S2	0.15066 (5)	1.01218 (9)	0.20597 (4)	0.0324 (2)	
O1	0.41835 (14)	1.0121 (3)	0.32740 (12)	0.0477 (7)	
O2	0.46882 (15)	0.9002 (3)	0.41908 (14)	0.0578 (8)	
H2D	0.5089	0.9490	0.4171	0.087*	
O3	0.05065 (14)	0.6603 (3)	0.32417 (13)	0.0496 (7)	
H3D	0.0114	0.6090	0.3250	0.074*	
O4	0.10411 (15)	0.5422 (3)	0.41363 (13)	0.0520 (7)	
O5	0.00331 (15)	1.0344 (3)	0.13061 (14)	0.0539 (8)	
O6	−0.05464 (15)	0.9000 (3)	0.04927 (14)	0.0506 (7)	
H6D	−0.0924	0.9549	0.0482	0.076*	
O7	0.30978 (15)	0.5421 (3)	0.05144 (13)	0.0442 (7)	
O8	0.36843 (15)	0.6784 (3)	0.13228 (15)	0.0591 (8)	
H8D	0.4087	0.6302	0.1299	0.089*	
C1	0.33165 (18)	0.8542 (3)	0.37059 (15)	0.0254 (7)	
C2	0.26309 (18)	0.8786 (3)	0.32073 (15)	0.0256 (7)	
C3	0.19173 (18)	0.8031 (3)	0.32198 (15)	0.0267 (7)	
H3A	0.1462	0.8166	0.2893	0.032*	
C4	0.18767 (18)	0.7073 (3)	0.37180 (15)	0.0267 (7)	
C5	0.25458 (19)	0.6852 (3)	0.42111 (15)	0.0292 (7)	
H5	0.2514	0.6215	0.4544	0.035*	
C6	0.32567 (19)	0.7586 (3)	0.42018 (15)	0.0305 (8)	
H6	0.3707	0.7443	0.4532	0.037*	
C7	0.41062 (19)	0.9292 (3)	0.37033 (17)	0.0331 (8)	
C8	0.11000 (19)	0.6282 (3)	0.37180 (16)	0.0306 (7)	
C9	0.15409 (19)	0.8884 (3)	0.14229 (16)	0.0289 (7)	
C10	0.08448 (18)	0.8614 (3)	0.09422 (15)	0.0292 (7)	
C11	0.0880 (2)	0.7598 (3)	0.04614 (17)	0.0365 (8)	
H11	0.0416	0.7418	0.0150	0.044*	
C12	0.1597 (2)	0.6863 (3)	0.04463 (17)	0.0344 (8)	
H12	0.1616	0.6190	0.0129	0.041*	
C13	0.22814 (19)	0.7144 (3)	0.09087 (16)	0.0301 (7)	
C14	0.22585 (18)	0.8129 (3)	0.13977 (16)	0.0300 (7)	
H14	0.2725	0.8287	0.1710	0.036*	
C15	0.0074 (2)	0.9392 (3)	0.09224 (17)	0.0331 (8)	
C16	0.30639 (19)	0.6367 (3)	0.08988 (16)	0.0319 (7)	

### Atomic displacement parameters (Å^2^)

**Table:**

	*U*^11^	*U*^22^	*U*^33^	*U*^12^	*U*^13^	*U*^23^
S1	0.0270 (4)	0.0334 (5)	0.0317 (5)	−0.0071 (3)	−0.0001 (3)	0.0045 (3)
S2	0.0282 (4)	0.0332 (5)	0.0347 (5)	0.0045 (4)	0.0013 (3)	−0.0017 (4)
O1	0.0276 (13)	0.0655 (18)	0.0462 (15)	−0.0226 (12)	−0.0062 (11)	0.0224 (14)
O2	0.0245 (14)	0.075 (2)	0.0660 (18)	−0.0225 (13)	−0.0174 (13)	0.0367 (15)
O3	0.0268 (14)	0.0661 (19)	0.0513 (16)	−0.0234 (12)	−0.0079 (12)	0.0185 (13)
O4	0.0292 (14)	0.0607 (17)	0.0616 (18)	−0.0227 (12)	−0.0072 (12)	0.0274 (14)
O5	0.0308 (14)	0.0606 (18)	0.0651 (18)	0.0206 (13)	−0.0094 (12)	−0.0262 (15)
O6	0.0270 (14)	0.0599 (18)	0.0594 (17)	0.0170 (12)	−0.0105 (12)	−0.0205 (14)
O7	0.0339 (14)	0.0468 (15)	0.0514 (16)	0.0141 (11)	0.0055 (12)	−0.0084 (12)
O8	0.0292 (15)	0.069 (2)	0.075 (2)	0.0203 (13)	−0.0069 (14)	−0.0258 (16)
C1	0.0186 (16)	0.0304 (17)	0.0271 (16)	−0.0088 (13)	0.0029 (12)	−0.0036 (13)
C2	0.0240 (16)	0.0262 (16)	0.0275 (17)	−0.0054 (13)	0.0066 (13)	−0.0019 (13)
C3	0.0200 (16)	0.0313 (17)	0.0277 (17)	−0.0069 (13)	−0.0001 (13)	−0.0014 (13)
C4	0.0213 (16)	0.0305 (17)	0.0289 (17)	−0.0072 (13)	0.0052 (13)	−0.0002 (13)
C5	0.0263 (17)	0.0335 (18)	0.0280 (17)	−0.0079 (14)	0.0050 (13)	0.0047 (13)
C6	0.0203 (17)	0.040 (2)	0.0294 (17)	−0.0068 (14)	−0.0039 (13)	0.0047 (14)
C7	0.0228 (17)	0.039 (2)	0.0361 (19)	−0.0104 (15)	0.0010 (14)	0.0021 (15)
C8	0.0226 (17)	0.0327 (19)	0.0366 (19)	−0.0100 (14)	0.0045 (14)	0.0016 (15)
C9	0.0255 (17)	0.0286 (17)	0.0328 (18)	0.0003 (13)	0.0051 (14)	0.0031 (14)
C10	0.0210 (17)	0.0333 (18)	0.0329 (18)	0.0030 (14)	0.0032 (13)	0.0008 (14)
C11	0.0252 (18)	0.044 (2)	0.038 (2)	0.0059 (15)	−0.0012 (15)	−0.0051 (16)
C12	0.0288 (18)	0.0374 (19)	0.0362 (19)	0.0068 (15)	0.0024 (15)	−0.0066 (15)
C13	0.0262 (18)	0.0309 (18)	0.0333 (18)	0.0062 (14)	0.0048 (14)	0.0025 (14)
C14	0.0186 (16)	0.0340 (18)	0.0361 (18)	0.0037 (13)	−0.0001 (14)	0.0028 (14)
C15	0.0248 (18)	0.0357 (19)	0.0377 (19)	0.0070 (14)	0.0012 (15)	−0.0027 (15)
C16	0.0242 (18)	0.0365 (19)	0.0348 (19)	0.0049 (14)	0.0042 (14)	0.0011 (15)

### Geometric parameters (Å, °)

**Table:**

S1—C2	1.795 (3)	C3—C4	1.394 (4)
S1—S2	2.0476 (11)	C3—H3A	0.9300
S2—C9	1.787 (3)	C4—C5	1.386 (4)
O1—C7	1.217 (4)	C4—C8	1.493 (4)
O2—C7	1.299 (4)	C5—C6	1.374 (4)
O2—H2D	0.8200	C5—H5	0.9300
O3—C8	1.303 (4)	C6—H6	0.9300
O3—H3D	0.8200	C9—C14	1.399 (4)
O4—C8	1.216 (4)	C9—C10	1.410 (4)
O5—C15	1.229 (4)	C10—C11	1.407 (4)
O6—C15	1.295 (4)	C10—C15	1.473 (4)
O6—H6D	0.8200	C11—C12	1.384 (4)
O7—C16	1.223 (4)	C11—H11	0.9300
O8—C16	1.296 (4)	C12—C13	1.378 (4)
O8—H8D	0.8200	C12—H12	0.9300
C1—C6	1.395 (4)	C13—C14	1.395 (4)
C1—C2	1.415 (4)	C13—C16	1.497 (4)
C1—C7	1.491 (4)	C14—H14	0.9300
C2—C3	1.390 (4)		
			
C2—S1—S2	104.58 (10)	O4—C8—O3	124.0 (3)
C9—S2—S1	103.87 (11)	O4—C8—C4	121.5 (3)
C7—O2—H2D	109.5	O3—C8—C4	114.4 (3)
C8—O3—H3D	109.5	C14—C9—C10	118.0 (3)
C15—O6—H6D	109.5	C14—C9—S2	120.6 (2)
C16—O8—H8D	109.5	C10—C9—S2	121.3 (2)
C6—C1—C2	119.9 (3)	C11—C10—C9	120.1 (3)
C6—C1—C7	119.6 (3)	C11—C10—C15	118.5 (3)
C2—C1—C7	120.5 (3)	C9—C10—C15	121.4 (3)
C3—C2—C1	118.3 (3)	C12—C11—C10	120.8 (3)
C3—C2—S1	121.0 (2)	C12—C11—H11	119.6
C1—C2—S1	120.7 (2)	C10—C11—H11	119.6
C2—C3—C4	120.6 (3)	C13—C12—C11	119.0 (3)
C2—C3—H3A	119.7	C13—C12—H12	120.5
C4—C3—H3A	119.7	C11—C12—H12	120.5
C5—C4—C3	120.8 (3)	C12—C13—C14	121.2 (3)
C5—C4—C8	119.9 (3)	C12—C13—C16	119.9 (3)
C3—C4—C8	119.3 (3)	C14—C13—C16	118.8 (3)
C6—C5—C4	119.1 (3)	C13—C14—C9	120.7 (3)
C6—C5—H5	120.5	C13—C14—H14	119.7
C4—C5—H5	120.5	C9—C14—H14	119.7
C5—C6—C1	121.3 (3)	O5—C15—O6	122.9 (3)
C5—C6—H6	119.4	O5—C15—C10	120.7 (3)
C1—C6—H6	119.4	O6—C15—C10	116.4 (3)
O1—C7—O2	123.5 (3)	O7—C16—O8	124.0 (3)
O1—C7—C1	121.4 (3)	O7—C16—C13	121.4 (3)
O2—C7—C1	115.1 (3)	O8—C16—C13	114.6 (3)
			
C2—S1—S2—C9	−88.20 (15)	S1—S2—C9—C14	1.9 (3)
C6—C1—C2—C3	−1.5 (4)	S1—S2—C9—C10	−179.6 (2)
C7—C1—C2—C3	178.1 (3)	C14—C9—C10—C11	0.9 (5)
C6—C1—C2—S1	176.3 (2)	S2—C9—C10—C11	−177.6 (3)
C7—C1—C2—S1	−4.1 (4)	C14—C9—C10—C15	−178.1 (3)
S2—S1—C2—C3	2.9 (3)	S2—C9—C10—C15	3.4 (4)
S2—S1—C2—C1	−174.9 (2)	C9—C10—C11—C12	−0.9 (5)
C1—C2—C3—C4	1.0 (5)	C15—C10—C11—C12	178.2 (3)
S1—C2—C3—C4	−176.9 (2)	C10—C11—C12—C13	−0.3 (5)
C2—C3—C4—C5	0.0 (5)	C11—C12—C13—C14	1.4 (5)
C2—C3—C4—C8	179.8 (3)	C11—C12—C13—C16	−179.7 (3)
C3—C4—C5—C6	−0.4 (5)	C12—C13—C14—C9	−1.4 (5)
C8—C4—C5—C6	179.8 (3)	C16—C13—C14—C9	179.7 (3)
C4—C5—C6—C1	−0.2 (5)	C10—C9—C14—C13	0.2 (5)
C2—C1—C6—C5	1.2 (5)	S2—C9—C14—C13	178.8 (2)
C7—C1—C6—C5	−178.4 (3)	C11—C10—C15—O5	−175.4 (3)
C6—C1—C7—O1	179.1 (3)	C9—C10—C15—O5	3.6 (5)
C2—C1—C7—O1	−0.5 (5)	C11—C10—C15—O6	5.8 (5)
C6—C1—C7—O2	−1.5 (5)	C9—C10—C15—O6	−175.2 (3)
C2—C1—C7—O2	178.9 (3)	C12—C13—C16—O7	−4.7 (5)
C5—C4—C8—O4	−1.4 (5)	C14—C13—C16—O7	174.2 (3)
C3—C4—C8—O4	178.8 (3)	C12—C13—C16—O8	175.0 (3)
C5—C4—C8—O3	178.2 (3)	C14—C13—C16—O8	−6.1 (5)
C3—C4—C8—O3	−1.6 (5)		

### Hydrogen-bond geometry (Å, °)

**Table:**

*D*—H···*A*	*D*—H	H···*A*	*D*···*A*	*D*—H···*A*
O8—H8D···O5^i^	0.82	1.81	2.632 (3)	174
O6—H6D···O7^ii^	0.82	1.83	2.633 (3)	166
O3—H3D···O1^iii^	0.82	1.81	2.623 (3)	174
O2—H2D···O4^iv^	0.82	1.82	2.639 (3)	174

Symmetry codes: (i) *x*+1/2, *y*−1/2, *z*; (ii) *x*−1/2, *y*+1/2, *z*; (iii) *x*−1/2, *y*−1/2, *z*; (iv) *x*+1/2, *y*+1/2, *z*.
